# Novel Model of Pulmonary Artery Banding Leading to Right Heart Failure in Rats

**DOI:** 10.1155/2015/753210

**Published:** 2015-10-04

**Authors:** Masataka Hirata, Daiki Ousaka, Sadahiko Arai, Michihiro Okuyama, Suguru Tarui, Junko Kobayashi, Shingo Kasahara, Shunji Sano

**Affiliations:** Departments of Cardiovascular Surgery, Okayama University Graduate School of Medicine, Dentistry, and Pharmaceutical Sciences, Okayama University Hospital, 2-5-1 Shikata-cho, Kita-ku, Okayama 700-8558, Japan

## Abstract

*Background*. Congenital heart diseases often involve chronic pressure overload of the right ventricle (RV) which is a major cause of RV dysfunction. Pulmonary artery (PA) banding has been used to produce animal models of RV dysfunction. We have devised a new and easier method of constricting the PA and compared it directly with the partial ligation method. *Methods*. Eight-week-old male Sprague-Dawley rats (240–260 g) were divided into three groups: sham operation, partial pulmonary artery ligation (PAL) procedure, and pulmonary artery half-closed clip (PAC) procedure. RV function and remodeling were determined by echocardiography and histomorphometry. *Results*. Surgical mortality was significantly lower in the PAC group while echocardiography revealed significantly more signs of RV dysfunction. At the 8th week after surgery RV fibrosis rate was significantly higher in the PAC group. *Conclusions*. This procedure of pulmonary artery banding in rats is easier and more efficient than partial ligation.

## 1. Introduction

The exact prevalence of pediatric heart failure is largely unknown but it is increasing. Recent advances in diagnostic methods, new surgical techniques, and improved perioperative management have contributed to an increased survival for patients with complex congenital heart disease who today often survive into adulthood [1].

Even after successful repair, however, right ventricle (RV) pressure overload remains in some patients and eventually impairs RV function and influences long-term mortality and morbidity [2–4].

Although compensated hypertrophy develops initially, ultimately RV failure will occur. The mechanisms underlying the progression from compensated to decompensated RV hypertrophy have not been well defined [5].

Clinically, the relationship between progressive fibrosis and RV function must be addressed [6, 7]. But studies of the mechanisms underlying the transition from a compensated state of hypertrophy to a decompensated state are difficult in patients, because invasive data cannot be easily obtained. For this purpose, animal models may be beneficial. Small experimental animals, such as rats, are widely used in cardiovascular research since they can provide a range of disease models, including cardiac hypertrophy and failure. A major advantage of these disease models is that cardiac material can be easily sampled to study the pathology of the disease in question.

The partial pulmonary artery ligation procedure has been widely used to produce right heart failure caused by pressure overload, but this procedure might have important drawbacks including a high surgical mortality caused by bleeding, sudden cardiac arrest, or pulmonary thrombus.

Pressure overload may also be induced using half-closed paper clips easily applied with a stopper. We could adjust the closing size by moving the stopper. The objective of this study was to establish rats model of RV failure using the pulmonary artery half-closed clip procedure comparing with partial ligation procedure.

## 2. Materials and Methods

### 2.1. Animal Care

All experimental procedures and protocols used in this investigation were reviewed and approved by the institutional animal care and use committee and were in accordance with the National Institutes of Health “guide for the care and use of laboratory animals” (National Institutes of Health publication number 85-23, revised 1996).

### 2.2. Animal Model

**Figure 1:**
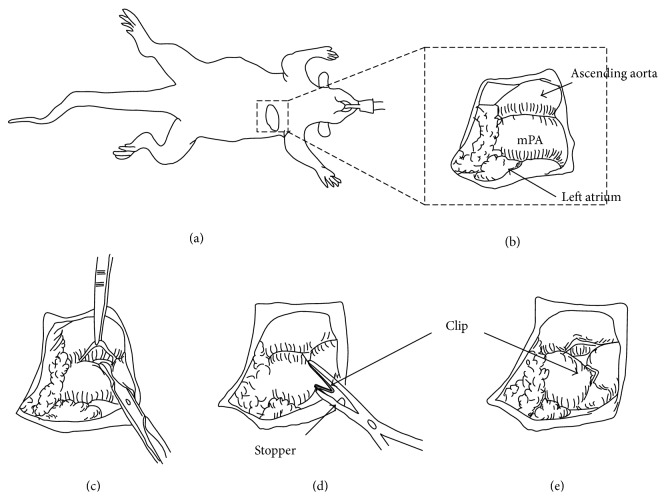
The PAC method. (a) Left thoracotomy was performed in the right semilateral decubitus position. (b) mPA was exposed through the left thoracotomy. ((c), (d)) Placement of a half-closed clip around mPA.

A rat model of the pulmonary artery banding (PAB) was established to create chronic RV pressure overload. Eight-week-old male Sprague-Dawley rats (240–260 g) were anesthetized with intraperitoneal pentobarbital (50 mg/kg body weight) and xylazine (5 mg/kg) and ventilated with 100% O_2_ by using a volume controlled respirator (2 mL, 60 cycles/min). A left thoracotomy was performed at the fourth intercostal space, and the main pulmonary artery (mPA) was carefully exposed (Figure 1).

#### 2.2.1. Partial Pulmonary Artery Ligation Model (PAL) (*n* = 28)

A 7-0 prolene suture was tied tightly around an 18-gauge needle alongside the mPA. After subsequent rapid removal of the needle, a fixed constricted opening was created in the lumen equal to the diameter of the needle [5, 8, 9].

#### 2.2.2. Half-Closed Clip Model (PAC) (*n* = 28)

**Figure 2:**
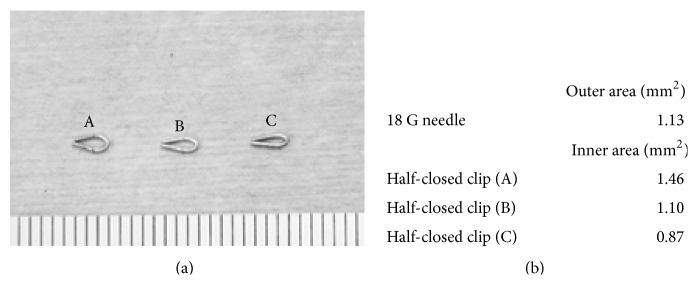
(a) Half-closed clips (A), (B), and (C) with different placed stopper. (b) Size of inner area of clips (A), (B), and (C), respectively, comparing with 18 G needle outer size. In this paper, we used (B) type for the PAC method.

A small clip (LT100 ETHICON) was half-closed around the mPA using a clip applier (LX107 ETHICON) with a stopper (Figure 1). The blood flow through the mPA was restricted to equal the inner segment of the half-closed clip. We selected type (B) clip because this inner size was almost equal to outer size of a 18 G needle (Figure 2).

Thereafter, the thorax was closed in layers, and the ventilator setting was changed (90 cycles/min) for half an hour to reduce the respiratory load.

#### 2.2.3. Sham Operation (*n* = 12)

A left thoracotomy was performed at the fourth intercostal space, and the mPA was carefully exposed. The thorax was closed in layers, and the ventilator setting was changed (90 cycles min^−1^) for half an hour to reduce the respiratory load.

### 2.3. Echocardiographic Measurements

Echocardiograms were recorded on the preoperative day and on the 4th week and 8th week after the procedure. The rats were anaesthetized for echos (pentobarbital 50 mg kg^−1^ body weight) but were breathing spontaneously and were positioned on their left side.

Measuring echocardiograms, the dose of anesthetics was reduced to 50% only on the day when a rat had developed clinical signs, loss of activity, body edema, and pleural effusion, of RV failure. Transthoracic 2-dimensional, M-mode (according to the standards of the American Society of Echocardiography) and Doppler imaging were performed with a 6.5 MHz transducer (Xario TOSHIBA, JAPAN).

**Figure 3:**
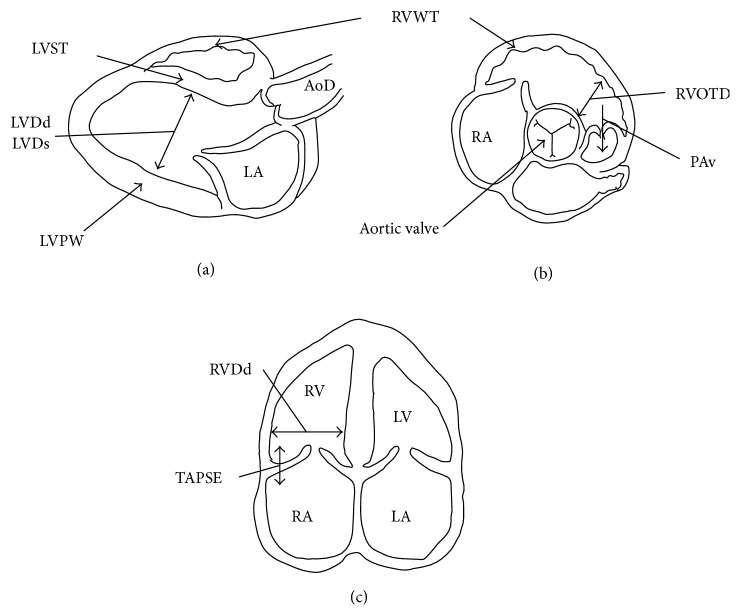
Schemata of echocardiograms measurements. (a) The parasternal long-axis view. (b) Parasternal short-axis view. (c) The apical four-chamber view.

Figure 3 showed schemata of the measurements of echocardiograms, respectively.

The RV morphology was assessed as RV free wall thickness (RVWT) and RV end-diastolic diameter (RVDd) and RV outflow tract dimension (RVOTD). RVWT was measured by M-mode either in the 2-dimensional parasternal short-axis view below the tricuspid valve or in the parasternal long-axis view, depending on the quality of RV free wall visualization. RVDd was measured as the maximal distance from the RV free wall to the septum from the apical four-chamber view.

RVOTD and aortic dimension (AoD) were measured at the aortic valve level in the short-axis view. The ratio of RVOTD to AoD (RVOTD/AoD) was calculated as representative of RV dilatation. The position of the transducer was aligned to visualize the RV apex.

The maximal RV cavity size preceding the frame with the onset of systolic closure of tricuspid valve leaflets was used to measure RVDd. To assess RV function, the tricuspid annular plane systolic excursion (TAPSE) of the lateral portion of the tricuspid annular plane was measured by the base-to-apex shortening during systole. This was recorded in the M-mode format under two-dimensional echocardiographic guidance from the apical four-chamber view.

Furthermore, PA velocity (PAv) was recorded at the aortic valve level in the short-axis view. This was used as an echocardiographic indicator of RV pressure overload [10, 11].

The left ventricle (LV) M-mode echocardiograms were recorded to evaluate LV function, as previously described. LV end-diastolic dimension (LVDd), LV end-systolic dimension (LVDs), interventricular septal wall thickness (IVST), and LV posterior wall thickness (LVPW) were measured at the papillary muscles level, and LV fractional shortening (LVFS) was calculated as follows:

(1) $$\begin{array}{c} LVFS=\left[\;\frac{\left(LVDd-LVDs\right)}{LVDd}\;\right]\times 100\left(\;\%\;\right). \end{array}$$

### 2.4. Histopathological Analysis

In the PAL group, the remaining 4 rats were killed 4 weeks after surgery and all the remaining rats (*n* = 8) were killed 8 weeks after surgery. In the PAC group, 4 rats were killed 4 weeks after surgery and all the remaining rats (*n* = 6) were killed 8 weeks after surgery. In the sham group, 6 rats were killed 4 or 8 weeks after surgery. The hearts were quickly removed, and the ventricles were dissected free of atrial tissue and large blood vessels. The right ventricle was carefully separated from the left ventricle and interventricular septum (IVS). The fresh ventricular tissues were immediately blotted dry and weighted separately to determine the degree of RV hypertrophy based on 2 parameters: RV wall weight/body weight (RV/BW) and RV wall weight/LV and IVS wall weight (RV/LV + IVS).

Tissue specimens for pathological analysis were obtained from whole hearts in cross sections, cut into 5 mm thick sections, and stained with hematoxylin and eosin for morphologic analysis, including measurement of the short-axis dimension of the RV myocardial cell and Masson's trichrome staining to determine the amount of interstitial and myocardial fibrosis. Digital images of cross sections were taken by a CCD camera (OLYMPUS DP-72) with a light microscope (OLYMPUS SZX12). The number of pixels of the blue-stained collagen area was calculated with Adobe Photoshop CS5 soft and then divided by the total number of pixels in the RV wall per slide, analyzed, and averaged.

### 2.5. Statistical Analysis

All data were expressed as mean ± SEM and range. Student's unpaired *t*-test or analysis of variance for parametric values was used to compare group means. Probability of 0.05 or less was considered to be statistically significant.

## 3. Results

### 3.1. Procedure

Surgical procedure time was defined as the interval from starting to incise the skin to skin closure. The surgical procedure time was 27.6 ± 1.4 min for PAL and 17.3 ± 2.1 min for PAC (*p* < 0.01). For the PAL procedure, 5 major hemorrhages occurred during trimming the mPA compared to only one major hemorrhage for the PAC procedure.

To evaluate the two procedures for RV pressure overload damage, we recorded the postsurgery recovery time. This was defined as the interval from time of extubation until the animal was fully conscious and walking freely. The recovery time was 30.4 ± 5.3 min for the sham procedure and 33.5 ± 5.2 min for PAC which was significantly shorter than the PAL procedure which was 64.9 ± 7.3 min.

### 3.2. Surgical Risk

Sixty-eight rats underwent PAL (*n* = 28) or PAC (*n* = 28) or sham (*n* = 12) in the surgical risk study. During the perisurgical period, which was the time from the beginning of surgery to 6 hours after surgery, 1 rat in the PAC group out of 28 died due to bleeding (3.6%).

In contrast, 7 of 28 perisurgical deaths were seen in the PAL group (25%). Of those 7 rats, 1 died from an intubation accident, 2 died from bleeding, 2 died from pneumothorax, and 2 died from sudden cardiac arrest. All of the animals in the sham group survived regardless of the surgical procedure. After the perisurgical period, there were 3 and 12 deaths in the PAL and PAC groups, respectively. The most common reasons for post-PAB death in both groups were pleural and/or pericardial effusion (pleural effusion: 66.7% in PAL and 83.3% in PAC, pericardial effusion: 33.3% in PAL and 41.7% in PAC) occurring between weeks 4 and 8 after surgery. Some of the rats died of unknown causes although we assumed these were due to either cardiac arrhythmia or heart failure as autopsy of these rats showed no blood in the thoracic cavity.

### 3.3. Echocardiographic Study

Table 1 shows the trend for LVDd and AoD to decrease but LV contractility (LVFS) showed no significant difference. Both PAL and PAC groups showed a significantly increased pulsed Doppler Peak PAv, RVOTD, RVDd, and RVWT. In the PAC group, PAv and RVOTD tended to increase more significantly than in the PAL group. The dilatation of the RV associated with the RVDd/LVDd ratio and the RVOTD/AoD ratio. In the PAC group, the RVDd/LVDd ratio was increased and became significantly greater than in the sham and PAL group. The RVOTD/AoD ratio was about 1.0 throughout the observation in the sham group. In contrast, the ratio increased progressively in the PAL and PAC groups, especially in the PAC group.

The signs of RV failure associated with TAPSE were more severe in the PAC group than in the PAL group. The signs of moderate or severe tricuspid regurgitation (TR) or pleural effusion or IVC dilation were detected in the PAC group 5 weeks after the PAC procedure, but we could seldom detect these signs in the PAL group throughout the observation period (PAC versus PAL: moderate or more TR 21/27 (77.8%) versus 3/21 (14.3%), pleural effusion 18/27 (66.7%) versus 2/21 (10.0%), and IVC dilation 20/27 (74.1%) versus 0/21 (0%)).

Furthermore, the standard deviation in the PAC group was greater than the PAL group. In other words, the data of the PAL group varied more widely than that of the PAC group. More stable data might be obtained with the PAC procedure.

### 3.4. Morphometric and Histological Analysis

#### 3.4.1. RV Hypertrophy

**Figure 4:**
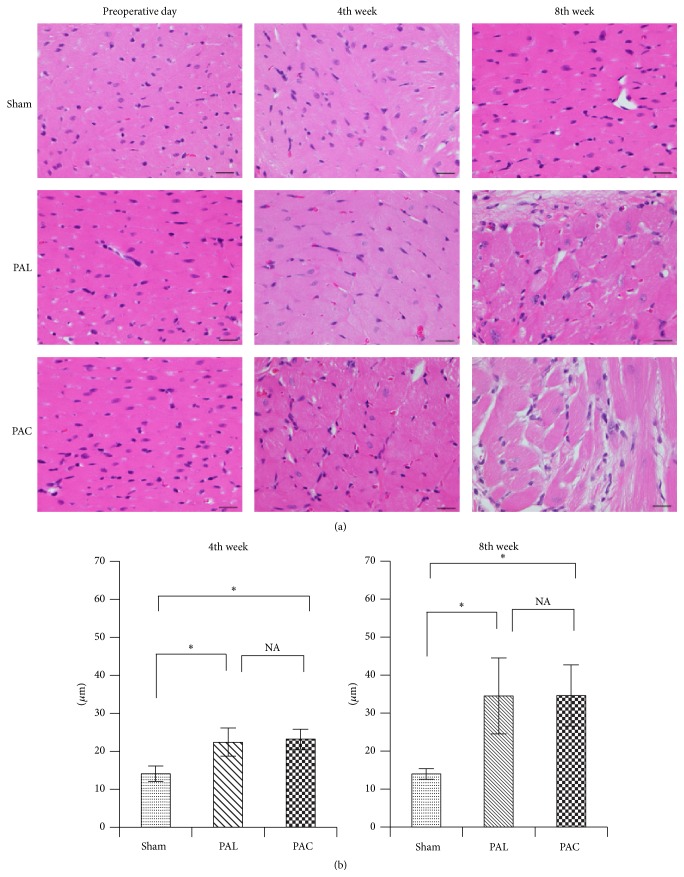
Photomicrographs of hematoxylin and eosin-stained sections showed significantly hypertrophied ventricular myocytes in the PAL and PAC groups. Scale bar: 20 *μ*m. ^*∗*^ *p* < 0.01.

**Figure 5:**
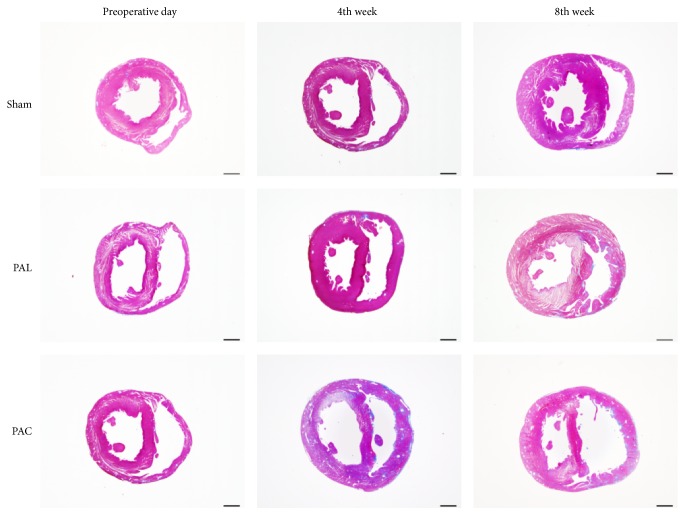
Macroscopic photographs of Masson's trichrome-stained cross sections showed RV wall thickening, cavity enlarging, and the interventricular septum shifting toward the left side in the PAL and PAC groups. Scale bar: 2 mm.

A weight analysis showed the heart weight/BW, RV/BW, and RV/(LV + IVS) weight ratios in the PAC and PAL groups to be similar and significantly higher than in the sham group (Table 2). Whole heart findings revealed a thickened RV wall, with an enlarged cavity, and the IVS shifted toward the left side in the PAC and PAL groups (Figure 5). Myocardial cell size in the PAC and PAL groups was similar and significantly higher than in the sham group (4th week: sham versus PAL versus PAC: 13.7 ± 1.64 *μ*m versus 21.3 ± 3.47 *μ*m versus 22.8 ± 2.38 *μ*m, 8th week: sham versus PAL versus PAC: 14.3 ± 1.40 *μ*m versus 34.7 ± 7.22 *μ*m versus 35.1 ± 8.19 *μ*m) (Table 1, Figure 4).

#### 3.4.2. Fibrosis

**Figure 6:**
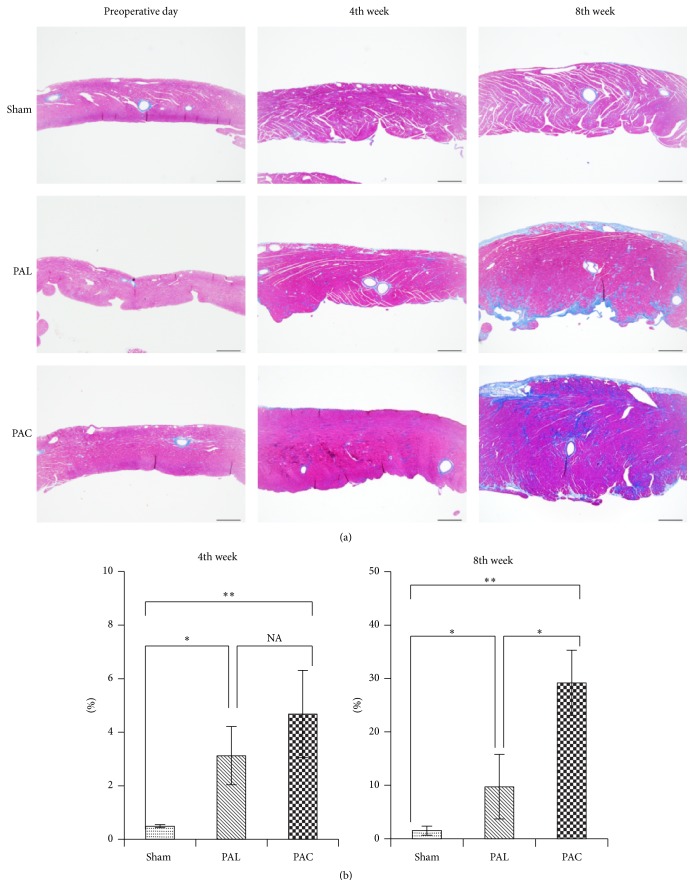
(a) Representative photomicrographs of Masson's trichrome-stained RV free wall (scale bar: 500 *μ*m). (b) Evaluation of fibrosis area for RV free wall. ^*∗*^ *p* < 0.05, ^*∗∗*^ *p* < 0.01.

Masson's trichrome staining showed fibrosis in the RV free walls of the sham, PAL, and PAC groups (Figure 6). At the 4th week, the percentage of fibrosis in the PAL and PAC groups was similar and significantly higher than in the sham group (sham versus PAL versus PAC: 0.49 ± 0.06% versus 3.12 ± 1.09% versus 4.68 ± 1.63%) (Figure 6(b)). But at 8 weeks, the percentage fibrosis in the PAC group was significantly higher than in the sham and PAL groups (sham versus PAL versus PAC: 1.51 ± 0.86% versus 9.73 ± 6.05% versus 29.2 ± 6.13%) (Figure 6(b)).

## 4. Discussion

Definition of heart failure was challenging because there were neither objective cutoff values of cardiac or ventricular dysfunction nor changes in pressure, dimension, or volume that could be reliably used to identify patients with heart failure. According to The European Society of Cardiology task force for the diagnosis and treatment of heart failure, both symptoms at rest or during exertion and objective evidence of cardiac dysfunction at rest should be present to diagnose heart failure [12].

In animal models, however, heart failure symptoms like fatigue or breathlessness are difficult to detect and quantify. Then evaluation of the models depends on objective findings such as reduced cardiac output, increased filling pressure, and progressive fibrosis [6, 7, 13]. In addition, the valuable signs of progressive heart failure are pericardial or pleural effusion [14]. In this paper, we demonstrated right heart failure by the objective findings of cardiac dysfunction.

Most attention is given to LV function, whereas RV function and disease have seldom been focused on. It is an established fact that there is a relationship between LV and RV function. Impairment of the RV might influence LV function [15, 16]. RV function is one independent predictor of mortality and the development of heart failure in patients with LV dysfunction [17]. Five to ten percent of patients with advanced chronic obstructive pulmonary disease may suffer from severe pulmonary hypertension and present with a progressively downhill clinical course because of RV dysfunction [18]. Not only in the view point of congenital heart disease, well-established animal models of RV function may be necessary for further investigation of RV function and pathology. Some animal models of RV dysfunction exist (monocrotaline treatment, partial ligation method) [5, 8–10, 19]. Monocrotaline treatment has been used to induce pulmonary hypertension resulting in RV hypertrophy and eventually heart failure [20, 21]. Monocrotaline treatment might have disadvantages in the form of disease manifestations not usually associated with human heart failure and changes in hormones such as endothelin [21, 22]. PAB does not have such side effects and is a promising procedure for inducing symptomatic RV dysfunction. PAL procedure has been used in several hypertrophy experiments; however, slippage of the band and the obvious difficulties ensuring the same degree of constriction among the banded animals when using a surgical nylon (usually prolene) led us to believe that banding with tantalum clips would be a more reliable procedure to create PAB model. In another PAB study using surgical nylon thread, PAL procedure, about 25% of the banded rats showed no hypertrophy [23], suggesting that this was a less reliable way. Also in this study, in the PAL group, the data had greater variance than in the PAC group. In addition, the objective findings of RV dysfunction were present less in the PAL group.

To the best of our knowledge this is the first reported evaluation of a newly devised means of stressing the right ventricle—the half-closed clip procedure—and its comparison with the pulmonary artery banding procedure. Using this new technique we could produce a right heart failure model in significantly less time and with much faster recovery and fewer surgical deaths than the PAL procedure. Most importantly, this novel procedure more fully developed subsequent myocardial damage and postsurgery cardiac dysfunction than did the PAL procedure. Furthermore, if we adjust the position of the stopper, we can produce different PAB models using this procedure.

A recent study in rats who underwent banding of the mPA by the existing partial ligation procedure showed that pressure overload alone was insufficient to explain right heart failure; the rats showed no effect on cardiac output or fibrosis [24]. Using the PAC procedure, however, we could produce RV dysfunction models that represent not only RV cavity dilation and RV hypertrophy but also moderate or greater tricuspid regurgitation or pleural and/or pericardial effusion.

Hardziyenka and colleagues reported LV atrophy in pulmonary hypertension [25]. However, we could not detect significant left-sided effects, but we could find a trend towards LV atrophy (Table 1 LVPWT, PAC versus sham, *p* = 0.051).

## 5. Conclusion

This study demonstrated that the application of a tantalum half-closed clip around the pulmonary artery induced right ventricle dysfunction in a reproducible manner. This model should prove valuable in the investigation and treatment of right heart failure caused by pressure overload.

## Figures and Tables

**Table 1:** Ultrasound findings in the sham rats, at 4th or 8th week after the PAB procedure.

	Preoperative day			4th week			8th week		
	Sham	PAL	PAC	Sham	PAL	PAC	Sham	PAL	PAC
*n*	12	28	28	12	16	22	6	8	6
LVDd (mm)	4.66 ± 0.43	4.75 ± 0.43	4.67 ± 0.39	5.36 ± 0.36	5.10 ± 0.42	5.07 ± 0.51	5.78 ± 0.19	5.40 ± 0.29	5.42 ± 0.38
LVDs (mm)	2.10 ± 0.5	2.15 ± 0.47	2.06 ± 0.47	2.51 ± 0.38	2.88 ± 0.58	2.55 ± 0.31	2.90 ± 0.52	2.38 ± 0.38	2.68 ± 0.45
IVST (mm)	1.06 ± 0.27	1.11 ± 0.29	1.14 ± 0.31	1.57 ± 0.10	1.56 ± 0.16	1.65 ± 0.27	1.93 ± 0.21	1.90 ± 0.10	2.13 ± 0.22
PWT (mm)	1.01 ± 0.24	1.05 ± 0.27	1.04 ± 0.27	1.71 ± 0.29	1.51 ± 0.08	1.62 ± 0.20	2.23 ± 0.22	2.04 ± 0.09	1.97 ± 0.14
FS (%)	55.17 ± 8.74	55.02 ± 8.17	56.15 ± 8.68	53.27 ± 4.42	42.81 ± 15.09	49.78 ± 4.02	49.62 ± 10.14	56.07 ± 5.10	50.64 ± 5.51
RVDd (mm)	2.46 ± 0.31	2.49 ± 0.29	2.49 ± 0.26	2.43 ± 0.28	**5.48 ± 1.47^∗^**	**6.26 ± 1.28^∗^**	2.80 ± 0.58	**5.26 ± 0.96^∗^**	**6.95 ± 0.63^∗^**
RVWT (mm)	0.63 ± 0.19	0.65 ± 0.18	0.70 ± 0.17	0.54 ± 0.08	**1.25 ± 0.11^∗^**	**1.41 ± 0.28^∗^**	0.98 ± 0.1	**1.72 ± 0.15^∗^**	**1.80 ± 0.43^∗^**
TAPSE (mm)	2.71 ± 0.22	2.69 ± 0.21	2.69 ± 0.23	2.53 ± 0.14	2.40 ± 0.11	**1.67 ± 0.20^∗^** ^$^	2.58 ± 0.1	2.32 ± 0.20	**1.42 ± 0.25^∗^** ^$^
HR (bpm)	438 ± 43	441 ± 39	455 ± 24	447 ± 21	436 ± 26	422 ± 31	451 ± 22	419 ± 20	411 ± 30
Peak PAV (m/s)	0.76 ± 0.11	0.73 ± 0.12	0.71 ± 0.12	0.97 ± 0.14	**2.93 ± 0.54^∗^**	**3.91 ± 0.38^∗^** ^$^	1.05 ± 0.13	**3.28 ± 0.37^∗^**	**4.03 ± 0.27^∗^** ^$^
RVOTD (mm)	1.99 ± 0.20	2.00 ± 0.24	1.97 ± 0.25	2.64 ± 0.17	**2.98 ± 0.37^#^**	**3.22 ± 0.50^∗^**	2.20 ± 0.16	**2.78 ± 0.37** ^#^	**3.62 ± 0.16^∗^** ^$^
AoD (mm)	2.23 ± 0.25	2.25 ± 0.23	2.30 ± 0.21	2.80 ± 0.18	2.65 ± 0.11	**2.55 ± 0.22^#^**	2.78 ± 0.05	2.44 ± 0.35	2.65 ± 0.10
RVDd/LVDd	0.53 ± 0.08	0.53 ± 0.07	0.53 ± 0.05	0.46 ± 0.07	**1.08 ± 0.28^∗^**	**1.25 ± 0.32^∗^**	0.48 ± 0.09	**0.971 ± 0.15^∗^**	**1.28 ± 0.08^∗^** ^$^
RVOTD/AoD	0.90 ± 0.16	0.90 ± 0.16	0.86 ± 0.15	0.95 ± 0.07	**1.12 ± 0.14^#^**	**1.27 ± 0.20^∗^**	0.79 ± 0.07	**1.157 ± 0.21^#^**	**1.37 ± 0.10^∗†^**
RVPW/LVPW	0.65 ± 0.23	0.65 ± 0.21	0.69 ± 0.20	0.32 ± 0.05	**0.83 ± 0.08^∗^**	**0.87 ± 0.15^∗^**	0.44 ± 0.06	**0.844 ± 0.07^∗^**	**0.92 ± 0.24^∗^**

*n*, number of rats. #: *p* < 0.05 versus sham, ∗: *p* < 0.01 versus sham, †: *p* < 0.05 versus PAL, and $: *p* < 0.01 versus PAL.

**Table:** (a) Weight analysis at the 4th week after the procedure

	BW	HW/BW	RV/BW	RV/(LV + IVS)
	(g)	ratio (mg/g)	ratio (mg/g)	ratio
Sham	435 ± 39	3.57 ± 0.16	0.56 ± 0.07	0.21 ± 0.02
PAL	403 ± 21	**4.65 ± 0.17^∗^**	**1.40 ± 0.13^∗^**	**0.50 ± 0.04^∗^**
PAC	398 ± 33	**4.88 ± 0.17^∗^**	**1.39 ± 0.03^∗^**	**0.49 ± 0.04^∗^**

**Table:** (b) Weight analysis at the 8th week after the procedure

	BW	HW/BW	RV/BW	RV/(LV + IVS)
	(g)	ratio (mg/g)	ratio (mg/g)	ratio
Sham	508 ± 41	3.30 ± 0.23	0.48 ± 0.04	0.21 ± 0.01
PAL	505 ± 47	**4.34 ± 0.30^∗^**	**1.27 ± 0.12^∗^**	**0.54 ± 0.02^∗^**
PAC	450 ± 50	**4.81 ± 0.32^∗^**	**1.40 ± 0.07^∗^**	**0.58 ± 0.03^∗^**

There was no significant difference between the PAL and PAC groups.

BW: body weight at the sacrifice time, HW: heart weight, and ∗: *p* < 0.05 versus sham.
